# Physical activity, sleep and risk of respiratory infections: A Swedish cohort study

**DOI:** 10.1371/journal.pone.0190270

**Published:** 2018-01-04

**Authors:** Francesca Ghilotti, Ann-Sofie Pesonen, Sara E. Raposo, Henric Winell, Olof Nyrén, Ylva Trolle Lagerros, Amelie Plymoth

**Affiliations:** 1 Department of Medicine, Clinical Epidemiology Unit, Karolinska Institutet, Stockholm, Sweden; 2 Department of Statistics and Quantitative Methods, University of Milano-Bicocca, Milan, Italy; 3 Department of Medical Epidemiology and Biostatistics, Karolinska Institutet, Stockholm, Sweden; 4 Department of Nutrition, Harvard T.H. Chan School of Public Health, Boston, Massachusetts, United States of America; 5 Department of Statistics, Uppsala University, Uppsala, Sweden; 6 Department of Medicine, Clinic of Endocrinology, Metabolism and Diabetes, Karolinska University Hospital Huddinge, Stockholm, Sweden; Kliniken der Stadt Köln gGmbH, GERMANY

## Abstract

**Objectives:**

Previous studies found higher levels of physical activity to be protective against infections and that short and long sleep negatively affects the immune response. However, these relationships remain debatable. We aimed to investigate if physical activity and sleep habits affect incidence of upper respiratory tract infections (URTI) in a prospective cohort study.

**Methods:**

A total of 2,038 adults aged 25–64 years served as a random sample of the gainfully employed population of an industrial town in Sweden. Physical activity and sleep habits were estimated through self-reported questionnaires. Physical activity was expressed as metabolic energy turnover hours per day. Sleep was assessed as number of hours slept per night and its perceived quality. URTI outcome was prospectively self-reported during a 9-month follow-up period. Associations of physical activity and sleep with URTI were estimated using hurdle regression models adjusted for potential confounders.

**Results:**

During 1,583 person-years 1,597 URTI occurred, resulting in an incidence of 1.01 infections/person-year (95% CI 0.96–1.06). The fitted regression models did not provide support for an association with physical activity or sleep habits. Factors positively associated with experiencing URTI were having children ≤ 6 years, female gender, higher education and treatment for allergy, asthma or lung cancer. Having children ≤ 6 years and female gender were related to a higher number of URTI among those experiencing URTI.

**Conclusions:**

We did not find any association between physical activity, sleep duration or sleep quality and the occurrence of upper respiratory tract infections in adult Swedish population.

## Introduction

Upper respiratory tract infections (URTI), typically expressed as common colds, constitute the most common reason for primary care visits in many Nordic countries [1]. It is also a leading cause of missed days of work; rhinitis and common cold have been shown to produce at least one day a year of absenteeism in 37% of Swedish workers [2]. Not only are common colds responsible for absenteeism, but they may also result in a loss of productivity among those who work while being sick [2,3]. Consequently, despite the generally trivial symptomatology, these common diseases pose a large burden on society and cause substantial costs. Still, little is known about risk factors for URTI.

The relationship between physical activity and incidence of URTI has been a matter of scientific debate. Some studies have shown that 60–90% of people who exercise regularly experience fewer colds than more sedentary peers [4]. A Swedish cohort study with retrospective self-reporting of URTI episodes showed that people with moderate to high physical activity had an 18% reduced occurrence of URTI, compared to people with low levels of physical activity [5]. Similarly, an American 12-week cohort study observed a 43% reduction in the number of sick-days due to URTI among subjects who were defined as physically active, compared to subjects who were not [6]. Several other studies have reported similar inverse associations [7–9]. In contrast, a large cohort study in Finland did not find an association between physical activity and the risk of common cold, [10] neither did a recent Cochrane systematic review of 11 trials [11]. Previous studies have been criticised for their lack of validated methods for measuring incidence of URTI and insufficient control of confounding [5,6,12].

Sleep duration has also been suggested to be associated with URTI and sleep disturbance has been found to be a risk factor for inflammation [13]. In two experimental studies, Prather [14] and Cohen [15] found that the incidence of common cold after experimental viral exposure was increased in subjects with shorter sleep duration. These findings were consistent with two recent observational studies [16,17]. However, to our knowledge, the effects of long sleep duration on infections are not well established. We therefore aimed to investigate if physical activity, sleep duration and sleep quality were independently associated with the incidence of URTI in a large prospective population-based cohort study using hurdle regression models, a rarely applied methodology appropriate for count data with excess zeros.

## Materials and methods

### Study design and population

This study was part of a prospective cohort (SWEDE-I: Studies of Work Environment and Disease Epidemiology-Infections) set up to investigate work-related risk factors for common viral infections [18]. In late August 2011, postal invitations were sent to 14,008 gainfully employed individuals (25–64 years) living in Eskilstuna municipality, Sweden. Invited individuals who answered in the first questionnaire that they were not currently working were excluded (n = 119).

The number of individuals who responded and fulfilled the inclusion criteria was 2,237, of whom 199 had missing information on the URTI outcome. Therefore, the final cohort used for data analysis in this study consisted of 2,038 men and women. The Regional Ethics Review Board in Stockholm, Sweden, approved the study (Regionala etikprövningsnämnden i Stockholm: Dnr 2011/360-31/2). Written-informed consent was received electronically or by mail from all participants in the study.

### Exposure definition

To obtain information on physical activity, sleep habits, and relevant covariates, the participants were asked to answer five questionnaires including data on demography, work environment, work tasks, commuting behaviour, contact patterns, family structure, health status and lifestyle factors. Four of the questionnaires were available in both a paper version and an electronic web version. For technical reasons, the fifth questionnaire—an interactive electronic form containing the physical activity self-reporting instrument [19,20]—was available only on the web.

Total energy expenditure during an ordinary 24-hours day was computed from time spent at work, leisure time and time spent sleeping. For time at work, a modified version of the validated Lagerros instrument was used (see Table in S1 Table) [20]. This instrument proceeds from nine predefined intensity levels expressed as metabolic energy turnover (MET)—the ratio of metabolic rate during work and metabolic rate while resting [21]. The participants reported the time spent on each level during a regular working day and each reported duration (in hours) was multiplied by the corresponding MET value. For leisure time activities the validated web questionnaire Active-Q was used (See Table in S2 Table) [22]. Finally, a MET value of 0.9 was assigned for sleeping hours [21]. Work, leisure time and sleep MET-hours per day (MET-h/d) components plus/minus a remainder term were summed up to total MET-h/d. The remainder term, with a corresponding MET value of 2, ensured that the total time sum up to 24 hours. MET-h/d was then subdivided into thirds.

Information on sleep habits was recorded through the following multiple-choice questions: “How many hours, approximately, do you usually sleep on an ordinary weekday?” (< 5/5/6/7/8/≥ 9) and “How do you usually sleep?” (Well/Quite well/Neither well nor bad/Quite bad/Bad). Sleep duration was recoded into three groups representing short sleep (5 hours or less), the reference group (6 or 7 hours), and long sleep (8 or more hours). Sleep quality was categorized in two groups merging the categories “Well” and “Quite well” versus the remaining.

To control for potential confounding, we adjusted for age (25–34, 35–44, 45–54, 55–64), sex, presence of children ≤ 6 years in the household (yes/no), smoking (smoking daily during the last month or no smoking), travel mode to work (alone or with a family member, together with other people, and different travel modes), education (none, elementary school, or junior secondary school, upper secondary, and postsecondary), body mass index (BMI) (< 18.5, 18.5–25, 25–30, ≥ 30 kg/m^2^), number of self-reported estimated close contacts per day (0–4, 5–9, 10–19, 20–29, 30–44, ≥ 45), received treatment for allergy, asthma or lung cancer (yes/no), and received treatment for immunodeficiency or transplantation (yes/no).

### Outcome definition

The participants were asked to self-report any onset of fever, upper respiratory tract infection, or gastroenteritis, immediately as they occurred, during a 9-month follow-up period between September and May. When the participants reported an infection, on a website or via telephone, [23] they also answered questions about their symptoms in an automated, tree-structured questionnaire. In addition, they were asked to take a nasal swab and send the sampled material to the department of virology at Karolinska University Hospital, to test for the 14 most common viruses by PCR [18]. To ensure high compliance, the participants received frequent reminders, monthly newsletters, and feedback such as their own test results on the study website, along with a short text that described the characteristics of the specific virus.

Incidence of infections was defined as the number of disease events per person during an individual follow-up period. This period ranged from 2 weeks after enrolment into the study until early discontinuation of follow-up or end of study (June 30^th^ 2012), whichever occurred first. A self-report and/or a nasal swab were considered to constitute a disease event, even if the nasal swab was negative. It has been shown that self-diagnosis of common colds is usually correct [10] and that false positive reporting is rare [24]. A negative PCR could either be due to suboptimal sampling technique, suboptimal timing, or an infection caused by another agent than the 14 viruses that were tested for in this study.

URTI was defined as a disease event with at least one of the following symptoms: cough, runny nose, or sore throat [25]. Events with missing answers to all three URTI symptoms, or events with missing answers to one or two of them in combination with a negative answer to the remaining URTI symptoms, were classified as missing URTI and therefore excluded from the analysis (n = 199). For descriptive purpose, individuals were categorized into three groups according to the number of URTI they reported (0, 1, 2–8 infections).

### Statistical methods

Multiple imputation of missing data was used to create and analyse 50 imputed datasets, on which all the forthcoming analyses are based on. Incomplete variables were imputed under fully conditional specification using the random forest-based MICE algorithm [26,27] assuming a missing at random mechanism. The imputation model for each incomplete variable included the outcome, exposures, potential confounders and auxiliary variables. The percentage of missing values across the outcome, exposures and potential confounders varied between 0 and 34%, leading to a total of 40% of the records being incomplete. The incomplete variables, including the outcome, were imputed using all other variables, complete as well as incomplete, and all available data was used, i.e., subjects with missing outcomes were included in the imputation. Prior to the main analysis all subjects with missing outcome were removed as recommended by Von Hippel [28].

As the distribution of URTI exhibited a large number of zeros, i.e., subjects experiencing no URTI event during the whole follow-up period, the association between the number of URTI and the two exposures, physical activity and sleep, respectively, was modelled using hurdle regression models [29].

The hurdle regression method has been widely applied in health economics and epidemiology [30–33], and it is particularly well-suited for modelling count data with excess zeros. The excess zeros are typically categorized as either structural zeros or sampling zeros, i.e., arising from not-at-risk or at-risk populations respectively. Here, the endpoint of interest, by design, contains no structural zeros since all participants are considered being at risk of URTI for the entire duration of the study. In this setting, the realization of an URTI represents a hurdle that has been crossed. The hurdle model consists of two components: a *zero-hurdle* part modelling a right-censored outcome variable indicating subjects with zero or at least one URTI, respectively, as well as a *positive counts* part modelling a truncated outcome variable representing the number of URTI among those experiencing URTI [34]. The binary zero-hurdle part, targeting the probability of experiencing zero URTI, was estimated using a right-censored Poisson model where all URTI counts larger than zero were censored. The positive counts part, modelling the mean number of non-zero URTI, was estimated using a left-truncated negative binomial model where all URTI counts were truncated at zero. This approach allows us to separately assess the underlying processes associated with experiencing URTI, or not, and the number of URTI among those experiencing URTI.

Statistical analyses were performed using R (version 3.3.1; The R Foundation for Statistical Computing, Vienna, Austria). Multiple imputation was performed using the R add-on package mice (version 2.25). The hurdle models were estimated using the R add-on package countreg (version 0.2–0). Goodness-of-fit was assessed using rootograms [35]. All statistical tests were two-sided, and *p*-values smaller than 0.05 were considered statistically significant.

## Results

**Fig 1:**
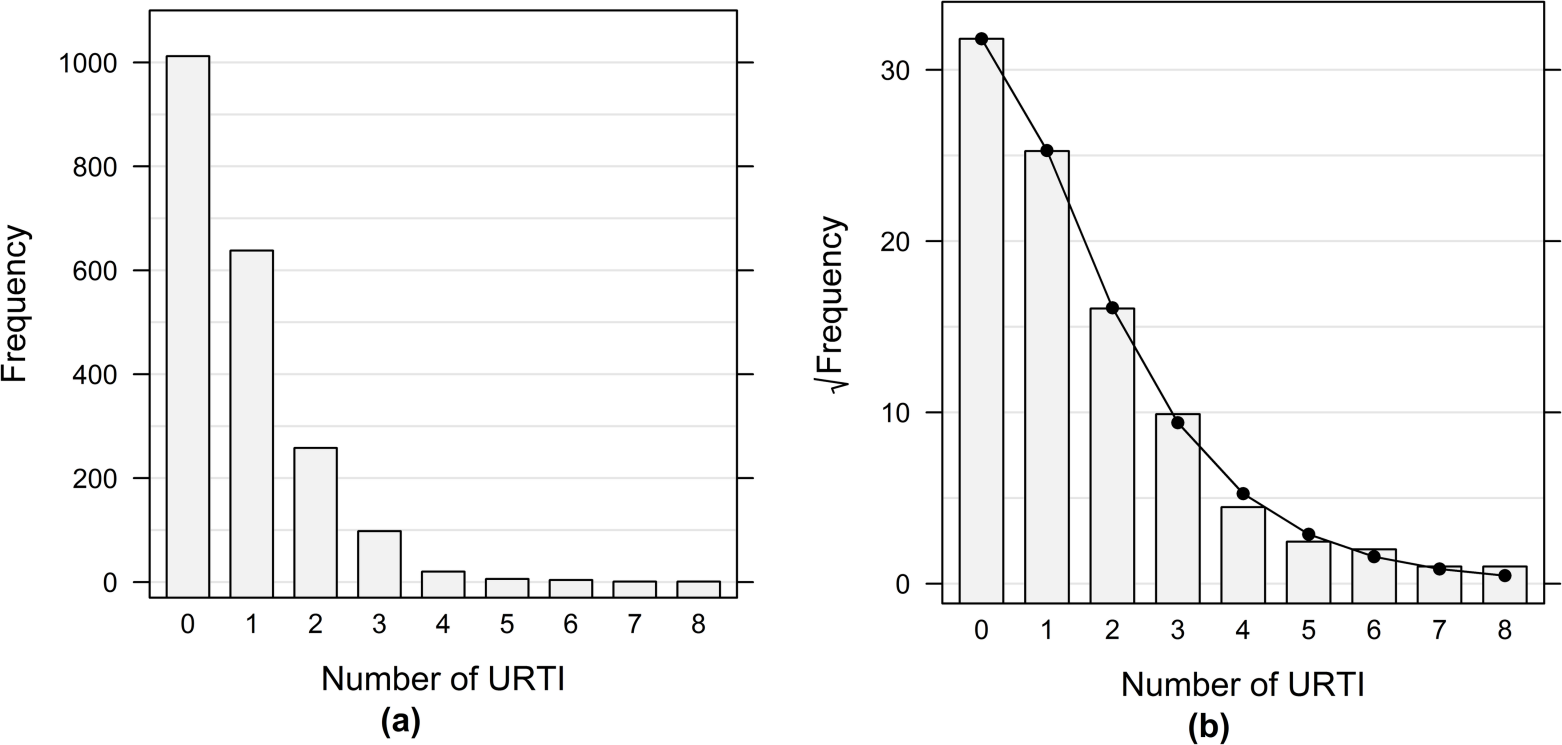
Frequency of URTI and goodness of fit. (a) The bars represent frequency of self-reported URTI events in the study population (N = 2,038). (b) Rootogram for model fit assessment of the MET-h/d model. Abbreviations: URTI = upper respiratory tract infections, MET-h/d = metabolic energy turnover–hours per day.

Half of the subjects included in the study did not report any URTI events, 31% reported one event, and 19% reported two or more events. The URTI frequency distribution is displayed in Fig 1A. Descriptive characteristics of the cohort among subjects with 0, 1 or 2–8 infections are shown in Table 1.

**Table 1:** Characteristics of study participants across groups of upper respiratory tract infections (URTI).

	Number of URTI^a^							
	0		1		2–8		Total	
Variable	(N = 1012)		(N = 638)		(N = 388)		(N = 2038)	
**Age (years)**								
25–34	12.0	(121)	13.8	(88)	15.2	(59)	13.2	(268)
35–44	31.4	(318)	31.4	(200)	36.9	(143)	32.4	(661)
45–54	28.1	(284)	26.3	(168)	23.7	(92)	26.7	(544)
55–64	28.5	(289)	28.5	(182)	24.2	(94)	27.7	(565)
**Sex**								
Women	52.5	(531)	63.0	(402)	71.1	(276)	59.3	(1209)
Men	47.5	(481)	37.0	(236)	28.9	(112)	40.7	(829)
**Children ≤ 6 years at home**								
No	84.5	(589)	82.0	(478)	74.5	(278)	81.4	(1345)
Yes	15.5	(108)	18.0	(105)	25.5	(95)	18.6	(308)
Missing		(315)		(55)		(15)		(385)
**Daily smoker**								
No	88.1	(614)	89.7	(525)	89.3	(334)	88.9	(1473)
Yes	11.9	(83)	10.3	(60)	10.7	(40)	11.1	(183)
Missing		(315)		(53)		(14)		(382)
**Mode of transport to work**								
Alone/With family member	89.5	(752)	89.5	(553)	90.3	(347)	89.7	(1652)
Together with others	9.4	(79)	9.5	(59)	9.4	(36)	9.4	(174)
Other ways	1.1	(9)	1.0	(6)	0.3	(1)	0.9	(16)
Missing		(172)		(20)		(4)		(196)
**Level of education**								
Post-secondary	51.7	(357)	60.2	(351)	67.4	(252)	58.2	(960)
Upper secondary	37.6	(260)	32.9	(192)	27.0	(101)	33.6	(553)
None/Elementary/Junior secondary	10.7	(74)	6.9	(40)	5.6	(21)	8.2	(135)
Missing		(321)		(55)		(14)		(390)
**Body mass index (kg/m**^**2**^**)**								
< 18.5	0.2	(1)	0.7	(4)	1.1	(4)	0.6	(9)
18.5–25.0	43.5	(290)	48.3	(273)	52.4	(185)	47.2	(748)
25.0–30.0	41.0	(273)	36.1	(204)	33.7	(119)	37.6	(596)
≥ 30.0	15.3	(102)	14.9	(84)	12.8	(45)	14.6	(231)
Missing		(346)		(73)		(35)		(454)
**Number of close contacts**								
< 5	11.1	(77)	8.7	(51)	8.3	(31)	9.6	(159)
5–9	17.6	(123)	21.1	(123)	16.4	(61)	18.5	(307)
10–19	28.3	(197)	27.9	(163)	28.4	(106)	28.2	(466)
20–29	16.2	(113)	18.7	(109)	20.1	(75)	18.0	(297)
30–44	14.2	(99)	12.5	(73)	13.1	(49)	13.4	(221)
≥ 45	12.6	(88)	11.1	(65)	13.7	(51)	12.3	(204)
Missing		(315)		(54)		(15)		(384)
**Treatment: Allergy/Asthma/Lung disease**								
No	80.3	(539)	75.4	(421)	73.2	(265)	77.0	(1225)
Yes	19.7	(132)	24.6	(137)	26.8	(97)	23.0	(366)
Missing		(341)		(80)		(26)		(447)
**Treatment: Immunodeficiency/Transplantation**								
No	97.9	(656)	98.6	(546)	98.3	(351)	98.2	(1553)
Yes	2.1	(14)	1.4	(8)	1.7	(6)	1.8	(28)
Missing		(342)		(84)		(31)		(457)
**Physical activity (MET-h/d)**								
< 37.7	33.9	(183)	33.4	(161)	32.6	(107)	33.4	(451)
37.7–43.9	31.7	(171)	33.0	(159)	36.3	(119)	33.3	(449)
≥ 43.9	34.4	(186)	33.6	(162)	31.1	(102)	33.3	(450)
Missing		(472)		(156)		(60)		(688)
**Sleep duration (hours)**								
≤ 5	7.8	(54)	7.8	(45)	4.0	(15)	7.0	(114)
6–7	77.2	(531)	74.6	(429)	79.9	(297)	76.9	(1257)
≥ 8	15.0	(103)	17.6	(101)	16.1	(60)	16.1	(264)
Missing		(324)		(63)		(16)		(403)
**Sleep quality**								
Quite good/Good	76.0	(525)	75.2	(433)	74.5	(277)	75.4	(1235)
Neither bad nor good/Quite bad/Bad	24.0	(166)	24.8	(143)	25.5	(95)	24.6	(404)
Missing	(321)		(62)		(16)		(399)	

^a^ The presented data is given as % (N)

**Fig 2:**
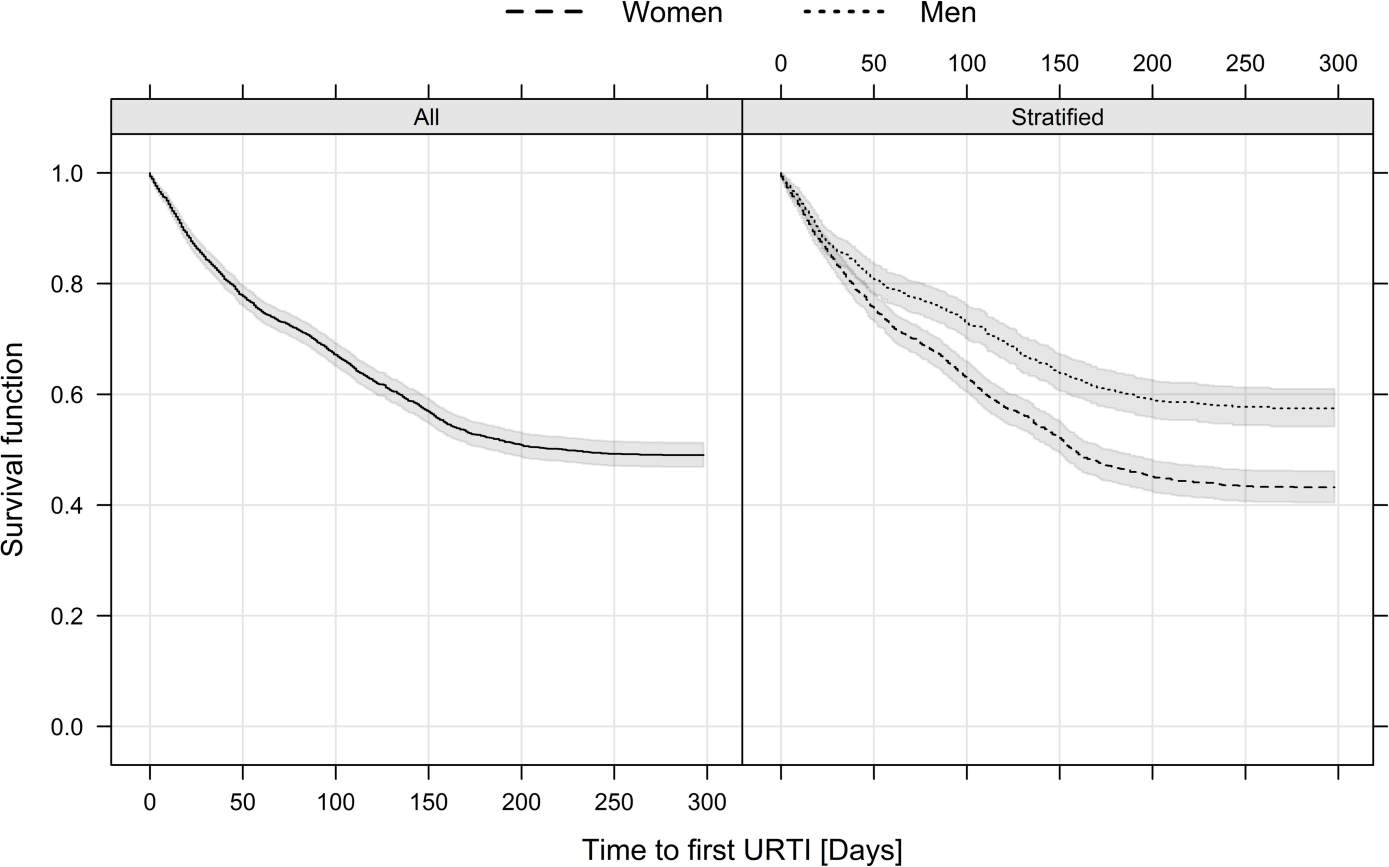
Kaplan-Meier plots illustrating the survival function of time to first URTI among all the subjects and stratified by sex. The shaded area is the (pointwise) 95% confidence interval. Abbreviations: URTI = upper respiratory tract infections.

In total, 1,876 infections occurred during 1,583 person-years; 1,597 out of 1,876 infections were URTI events, resulting in an incidence of 1.01 URTI/person-year (95% CI 0.96–1.06). The incidence rate of URTI was higher in women (1.17 URTI/person-year 95% CI 1.10–1.24) compared to men (0.77 URTI/person-year 95% CI 0.71–0.85). The median time until first URTI event was 222 days (95% CI 183-infinity) in the whole cohort and 158 days (95% CI 147–178) for women. Since less than 50% of the men had at least one URTI event, their median time was not defined. Kaplan-Meier plots illustrating the survival function of time to first URTI (overall and stratified by sex) with 95% confidence intervals are shown in Fig 2. One third of the subjects included in the cohort had a physical activity level below 37.7 MET-h/d, while one third had a level above 43.9 MET-h/d. Most of the subjects reported 6 or 7 hours of sleep per night, 7.0% slept 5 hours or less and 16.0% slept 8 hours or more. In addition, the majority reported a good quality of sleep. Among subjects sleeping 5 hours or less, only 30% reported good quality, while among participants sleeping 8 hours or more, 88% reported good quality.

Table 2 contains crude and adjusted URTI incidence rate ratios (IRRs), with 95% confidence intervals for physical activity (see also Table in S3 Table).

**Table 2:** Crude and adjusted URTI incidence rate ratio (IRR) estimates by level of physical activity and studied covariates, fitted in a hurdle regression model.

	Crude						Adjusted					
	Positive Counts			Zero-hurdle			Positive Counts			Zero-hurdle		
	IRR	95% CI	*p*-value	IRR	95% CI	*p*-value	IRR	95% CI	*p*-value	IRR	95% CI	*p*-value
**Physical activity (MET-h/d)**			0.17			0.87			0.46			1.00
< 37.7	Ref			Ref			Ref			Ref		
37.7–43.9	1.12	0.89–1.42		1.03	0.87–1.23		1.05	0.83–1.31		1.00	0.84–1.20	
≥ 43.9	0.90	0.70–1.14		0.99	0.83–1.18		0.91	0.71–1.15		1.01	0.84–1.21	

Physical activity did not exhibit any inverse association with URTI events, neither in the positive counts part (*p*-value 0.46) nor in the zero-hurdle one (*p*-value 1.00). Subjects whose physical activity level was between 37.7 and 43.9 MET-h/d or above 43.9 MET-h/d had about the same occurrence of one or more URTI as the subjects in the lowest third. The estimated incidence rate ratios (IRRs) were 1.00 (95% CI 0.84–1.20) and 1.01 (95% CI 0.84–1.21), respectively. Furthermore, for those experiencing URTI, the mean number of URTI among the subjects in the middle and highest physical activity level did not differ appreciably from the ones in the lowest third with IRRs of 1.05 (95% CI 0.83–1.31) and 0.91 (95% CI 0.71–1.15), respectively. The rootogram in Fig 1B indicates an excellent model fit that captures the underlying distribution with only minor deviations.

Sleep duration and sleep quality were neither associated with the probability of at all experiencing an URTI during follow-up (*p*-value 0.37 and 0.08, respectively), nor with the number of URTI among those who did (*p*-value 0.29 and 0.26, respectively) (Table 3, see also Table in S4 Table).

**Table 3:** Crude and adjusted URTI incidence rate ratio (IRR) estimates by level of sleep duration, sleep quality and studied covariates, fitted in a hurdle regression model.

	Crude						Adjusted					
	Positive Counts			Zero-hurdle			Positive Counts			Zero-hurdle		
	IRR	95% CI	*p*-value	IRR	95% CI	*p*-value	IRR	95% CI	*p*-value	IRR	95% CI	*p*-value
**Sleep duration (hours)**			0.25			0.06			0.29			0.37
6–7	Ref			Ref			Ref			Ref		
≤ 5	0.71	0.46–1.10		0.92	0.69–1.22		0.79	0.51–1.20		0.96	0.72–1.29	
≥ 8	0.90	0.70–1.17		1.23	1.02–1.48		0.86	0.67–1.11		1.14	0.94–1.38	
**Sleep quality**			0.30			0.03			0.26			0.08
Quite good/Good	Ref			Ref			Ref			Ref		
Neither bad nor good/Quite bad/Bad	1.12	0.90–1.40		1.20	1.02–1.41		1.13	0.92–1.39		1.16	0.98–1.37	

Subjects sleeping ≤ 5 or ≥ 8 hours had about the same occurrence of one or more URTI as the subjects sleeping 6 or 7 hours. The estimated incidence rate ratios (IRRs) were 0.96 (95% CI 0.72–1.29) and 1.14 (95% CI 0.94–1.38), respectively. Additionally, for those experiencing URTI, the mean number of URTI events among the subjects in the lowest and highest sleep category did not differ significantly from the ones in the reference category with IRRs of 0.79 (95% CI 0.51–1.20) and 0.86 (95% CI 0.67–1.11), respectively. The URTI incidence among subjects who do not sleep well was not statistically different from the ones with good sleep quality (IRR 1.13, 95% CI 0.92–1.39). Likewise, for subjects experiencing URTI, the mean number of URTI events among those with bad sleep quality was not statistically different from the ones with good sleep quality (IRR 1.16, 95% CI 0.98–1.37). To test for effect modification of sleep quality, a cross-product term of sleep duration and sleep quality was entered into the model. The *p*-value of the test for effect modification was 0.57 in the positive counts part and 0.69 in the zero-hurdle part, suggesting that sleep quality was not behaving as an effect modifier in the relationship between sleep duration and incidence of URTI.

Covariates positively associated with experiencing one or more URTI events were female gender, children ≤ 6 years in the family, a higher level of education, and treatment for allergy, asthma, or lung cancer. Having children ≤ 6 years and being female were also related to a higher number of URTI among those experiencing URTI (See Tables in S3 Table and S4 Table).

## Discussion

In this prospective cohort study, no evidence of an association between self-reported physical activity or self-reported sleep habits and the incidence of URTI was found. Our null result on physical activity agrees with some previous studies, including a Cochrane systematic review published in 2015, [11] but it disagrees with several observational studies reporting inverse associations between moderate physical activity and URTI incidence [5–9].

Not only sleep deprivation may negatively affect the immune response, [36] but also long sleep duration and bad sleep quality have been found to be associated with the immune profile measured by CD4 and cytokines [13]. Our results, however, did not confirm our original hypothesis of an increased susceptibility to infections in subjects whose sleep duration deviates from the reference (6–7 hours). This conclusion on sleep duration contrasts with two previous observational studies [16,17] as well as with two experimental studies [14,15].

Strengths of our study include the large and population-based sample, the long follow-up spanning from September through May, the prospective self-reporting of the outcome using a well-assessed methodology, and the richness of background information that permitted comprehensive control of potential confounding. In addition, the use of hurdle models allowed us to properly take into account the positively skewed URTI distribution and its excess of zero values [37].

Physical activity was self-reported and not objectively measured. Although we used validated instruments [20,22], a certain degree of exposure misclassification is unavoidable. People with low education and high BMI tend to overestimate their physical activity level [20,38]. To balance the impact of the latter misclassification we adjusted for education and BMI in our modelling. Sleep duration was also self-reported, but it has been shown that the average sleep obtained from 7-day diaries correlates well with the one collected from one single question about usual sleep duration [17].

A prospective cohort design with self-reporting of “typical” exposure level only at baseline is a safeguard against reversed causation. However, to avoid overloading the participants, the questionnaires were sent out one by one at approximately one-month intervals over the first half of the follow-up period. Therefore, we cannot exclude the possibility that the outcome sometimes affected the exposure reports. Moreover, if the physical activity pattern is variable over time and the hypothesized effect is short-lived, undocumented changes in physical activity level during the 9-month follow-up period may potentially have affected URTI incidence. On the other hand, occupational physical activity is likely fairly stable over time for most individuals.

A limitation may be underreporting of outcome. A validation study that examined self-initiated, event-driven infectious disease reporting noted that participants failed to report approximately 60% of the disease episodes [24]. This underreporting was remarkably stable over time, across seasons, and cohorts. It was also reasonably similar in strata of background factors, although men, elderly people, and subjects with low education were somewhat poorer reporters than others. In the present study, individuals who reported more infections had fewer missing answers in the exposure questionnaires, confirming that people with generally poorer compliance also exhibited lower URTI incidence rates. Since there is virtually no false positive reporting, the risk-ratio estimations should remain unbiased as long as the underreporting is non-differential among exposed and unexposed [39]. While underreporting was shown to be linked to male sex, age, and education, [24] we have less reason to believe that it was importantly linked to level of physical activity at work or to sleep habits.

A final weakness is that we only counted discrete episodes of URTI and did not follow their further course. Thus, if physical activity or sleep would be associated with a milder course or shorter duration of the episodes, possibly reflected by fewer days with URTI symptoms, this would not be captured in the present investigation, neither would a seasonal variation in the effects of our exposures on the outcome.

In conclusion, we did not find evidence of a protective effect of moderately intense physical activity or adequate sleep duration on the occurrence of upper respiratory tract infections in adult Swedish men and women. Beyond that, we proposed the use of hurdle regression models in a research field that might benefit from this rarely applied methodology. While this study has gone further than most other observational studies to remedy criticized weaknesses, other limitations preclude the firm rejection of the hypothesis.

## Supporting information

S1 Table The physical activity instrument used for self-reported physical activity at work. (DOCX)

S2 Table Questions included in Active-Q and corresponding MET values. (DOCX)

S3 Table Crude and adjusted URTI incidence rate ratio (IRR) estimates by level of physical activity and studied covariates, fitted in a hurdle regression model. (DOCX)

S4 Table Crude and adjusted URTI incidence rate ratio (IRR) estimates by level of sleep duration, sleep quality and studied covariates, fitted in a hurdle regression model. (DOCX)
