# Supplementary material for: Physical activity, sleep and risk of respiratory infections: A Swedish cohort study
Source: PLoS One. 2018 Jan 4;13(1):e0190270. doi: 10.1371/journal.pone.0190270 (PMC5754073; doi:10.1371/journal.pone.0190270)
Supplement: S1 Table — (DOCX) [file pone.0190270.s001.docx]

| **How long is your normal working day?** | | | |
| --- | --- | --- | --- |
| **Level** | **How many hours and minutes of your normal working day do you engage in activities that require the effort similar to…** | Hours | Minutes |
| **A** | for example sleeping, lying quietly in bed |  |  |
| **B** | for example sitting - bathing, quietly listening to music, watching television, etc. |  |  |
| **C** | for example sitting – light office work, knitting, sewing, meetings, etc. |  |  |
| **D** | for example making bed, ironing, washing dishes, etc. |  |  |
| **E** | for example bowling, driving bus/tractor, automobile repair, dancing waltz/foxtrot, etc. |  |  |
| **F** | for example walking briskly, horseback riding, sweeping sidewalk, etc. |  |  |
| **G** | for example painting outside house, carrying and stacking wood, skiing downhill, etc. |  |  |
| **H** | for example construction work, mowing lawn with hand mower, shoveling snow by hand, etc. |  |  |
| **I** | more effort than level H |  |  |
| Make sure that the sum is equivalent to the length of your normal working day | | | |
