# Supplementary material for: Physical activity, sleep and risk of respiratory infections: A Swedish cohort study
Source: PLoS One. 2018 Jan 4;13(1):e0190270. doi: 10.1371/journal.pone.0190270 (PMC5754073; doi:10.1371/journal.pone.0190270)
Supplement: S2 Table — (DOCX) [file pone.0190270.s002.docx]

| **Activity category** |  | **MET value** |
| --- | --- | --- |
| Daily occupation |  |  |
|  | Mostly sitting | 1.5 |
|  | A combination of sitting and standing up | 2.3 |
|  | Mostly standing up | 3.0 |
|  | Some physical activity | 4.5 |
|  | Heavy manual labor | 6.0 |
|  |  |  |
| Transportation |  |  |
|  | Walking | 4.0 |
|  | Bicycling | 4.0 |
|  | By motorcycle or scooter | 2.5 |
|  | By car or taxi | 1.0 |
|  | By bus, train, subway, or boat | 1.0 |
|  |  |  |
| Leisure time activities |  |  |
|  | Watching TV/DVDs | 1.0 |
|  | Using the computer | 1.0 |
|  | Sitting listening to music, sewing | 1.0 |
|  | Playing a musical instrument or active computer games | 2.0 |
|  | Doing household chores | 3.0 |
|  | Shopping or other errands | 2.3 |
|  | Dancing | 3.0 |
|  | Walking | 3.4 |
|  | Bicycling | 8.0 |
|  |  |  |
| Regular sporting activities |  |  |
|  | Aerobics | 6.5 |
|  | Weight lifting | 6 |
|  | Jogging or running | 8 |
|  | Athletics | 6 |
|  | Spinning | 8.5 |
|  | Swimming | 6 |
|  | Soccer, basketball, volleyball, or floorball (floor hockey) | 6 |
|  | Golf | 4.5 |
|  | Dance class | 4.5 |
|  | Horseback riding | 4 |
|  | Ice skating, ice hockey, or bandy | 7 |
|  | Skiing (downhill or cross country) | 7 |
|  | Martial arts | 10 |
|  | Boxing or wrestling | 6 |
|  | Tennis, badminton, or squash | 7 |
|  | Table tennis | 4 |
|  | Rowing, canoeing, surfing, or sailing | 3 |
|  | Motor sports | 4 |
|  | Rock climbing | 8 |
|  | Other | 2.5 |
