# Supplementary material for: Physical activity, sleep and risk of respiratory infections: A Swedish cohort study
Source: PLoS One. 2018 Jan 4;13(1):e0190270. doi: 10.1371/journal.pone.0190270 (PMC5754073; doi:10.1371/journal.pone.0190270)
Supplement: S4 Table — (DOCX) [file pone.0190270.s004.docx]

|  | **Crude** | | | | | | |  | **Adjusted** | | | | | | |
| --- | --- | --- | --- | --- | --- | --- | --- | --- | --- | --- | --- | --- | --- | --- | --- |
|  | **Positive Counts** | | |  | **Zero-hurdle** | | |  | **Positive Counts** | | |  | **Zero-hurdle** | | |
| Covariate | IRR | 95% CI | *p*-value |  | IRR | 95% CI | *p*-value |  | IRR | 95% CI | *p*-value |  | IRR | 95% CI | *p*-value |
| **Sleep duration (hours)** |  |  | 0.25 |  |  |  | 0.06 |  |  |  | 0.29 |  |  |  | 0.37 |
| 6-7 | Ref |  |  |  | Ref |  |  |  | Ref |  |  |  | Ref |  |  |
| ≤ 5 | 0.71 | 0.46-1.10 |  |  | 0.92 | 0.69-1.22 |  |  | 0.79 | 0.51-1.20 |  |  | 0.96 | 0.72-1.29 |  |
| ≥ 8 | 0.90 | 0.70-1.17 |  |  | 1.23 | 1.02-1.48 |  |  | 0.86 | 0.67-1.11 |  |  | 1.14 | 0.94-1.38 |  |
| **Sleep quality** |  |  | 0.30 |  |  |  | 0.03 |  |  |  | 0.26 |  |  |  | 0.08 |
| Quite good/Good | Ref |  |  |  | Ref |  |  |  | Ref |  |  |  | Ref |  |  |
| Neither bad nor good/Quite bad/Bad | 1.12 | 0.90-1.40 |  |  | 1.20 | 1.02-1.41 |  |  | 1.13 | 0.92-1.39 |  |  | 1.16 | 0.98-1.37 |  |
| **Age (years)** |  |  | < 0.01 |  |  |  | 0.16 |  |  |  | 0.42 |  |  |  | 0.81 |
| 35-44 | Ref |  |  |  | Ref |  |  |  | Ref |  |  |  | Ref |  |  |
| 25-34 | 0.98 | 0.75-1.28 |  |  | 1.07 | 0.88-1.31 |  |  | 0.97 | 0.75-1.25 |  |  | 1.08 | 0.88-1.33 |  |
| 45-54 | 0.72 | 0.56-0.91 |  |  | 0.88 | 0.75-1.04 |  |  | 0.82 | 0.64-1.06 |  |  | 1.00 | 0.84-1.20 |  |
| 55-64 | 0.72 | 0.56-0.91 |  |  | 0.90 | 0.76-1.06 |  |  | 0.84 | 0.65-1.09 |  |  | 1.06 | 0.89-1.27 |  |
| **Sex** |  |  | < 0.01 |  |  |  | < 0.01 |  |  |  | < 0.01 |  |  |  | < 0.01 |
| Women | Ref |  |  |  | Ref |  |  |  | Ref |  |  |  | Ref |  |  |
| Men | 0.71 | 0.58-0.87 |  |  | 0.66 | 0.58-0.75 |  |  | 0.75 | 0.61-0.92 |  |  | 0.70 | 0.61-0.80 |  |
| **Children ≤ 6 years at home** |  |  | < 0.01 |  |  |  | < 0.01 |  |  |  | < 0.01 |  |  |  | < 0.01 |
| No | Ref |  |  |  | Ref |  |  |  | Ref |  |  |  | Ref |  |  |
| Yes | 1.55 | 1.26-1.90 |  |  | 1.43 | 1.21-1.68 |  |  | 1.35 | 1.07-1.69 |  |  | 1.33 | 1.11-1.60 |  |
| **Daily smoker** |  |  | 0.41 |  |  |  | 0.86 |  |  |  | 0.75 |  |  |  | 0.72 |
| No | Ref |  |  |  | Ref |  |  |  | Ref |  |  |  | Ref |  |  |
| Yes | 0.87 | 0.63-1.20 |  |  | 1.02 | 0.82-1.27 |  |  | 0.95 | 0.70-1.30 |  |  | 1.04 | 0.83-1.30 |  |
| **Mode of transport to work** |  |  | 0.41 |  |  |  | 0.65 |  |  |  | 0.49 |  |  |  | 0.47 |
| Alone/With family member | Ref |  |  |  | Ref |  |  |  | Ref |  |  |  | Ref |  |  |
| Together with others | 0.95 | 0.69-1.31 |  |  | 1.07 | 0.86-1.33 |  |  | 1.01 | 0.74-1.37 |  |  | 1.15 | 0.92-1.44 |  |
| Other ways | 0.26 | 0.04-1.98 |  |  | 0.76 | 0.35-1.63 |  |  | 0.30 | 0.04-2.17 |  |  | 0.95 | 0.44-2.06 |  |
| **Level of education** |  |  | < 0.01 |  |  |  | < 0.01 |  |  |  | 0.24 |  |  |  | < 0.01 |
| Post-secondary | Ref |  |  |  | Ref |  |  |  | Ref |  |  |  | Ref |  |  |
| Upper secondary | 0.71 | 0.57-0.87 |  |  | 0.75 | 0.64-0.87 |  |  | 0.83 | 0.67-1.03 |  |  | 0.81 | 0.69-0.95 |  |
| None/Elementary/Junior secondary | 0.72 | 0.48-1.10 |  |  | 0.67 | 0.51-0.88 |  |  | 0.89 | 0.59-1.33 |  |  | 0.74 | 0.56-0.98 |  |
| **Body mass index (kg/m^2^)** |  |  | 0.43 |  |  |  | 0.09 |  |  |  | 0.90 |  |  |  | 0.66 |
| 18.5-25.0 | Ref |  |  |  | Ref |  |  |  | Ref |  |  |  | Ref |  |  |
| < 18.5 | 1.55 | 0.68-3.50 |  |  | 1.35 | 0.55-3.36 |  |  | 1.19 | 0.56-2.54 |  |  | 1.35 | 0.54-3.36 |  |
| 25.0-30.0 | 0.90 | 0.73-1.10 |  |  | 0.82 | 0.70-0.97 |  |  | 0.96 | 0.78-1.17 |  |  | 0.92 | 0.79-1.09 |  |
| ≥ 30.0 | 0.89 | 0.67-1.19 |  |  | 0.86 | 0.70-1.07 |  |  | 0.94 | 0.71-1.24 |  |  | 0.93 | 0.75-1.16 |  |
| **Number of close contacts** |  |  | 0.10 |  |  |  | 0.54 |  |  |  | 0.23 |  |  |  | 0.68 |
| 10-19 | Ref |  |  |  | Ref |  |  |  | Ref |  |  |  | Ref |  |  |
| < 5 | 0.83 | 0.57-1.22 |  |  | 0.94 | 0.73-1.22 |  |  | 0.89 | 0.61-1.28 |  |  | 0.97 | 0.75-1.26 |  |
| 5-9 | 0.83 | 0.63-1.11 |  |  | 1.10 | 0.90-1.34 |  |  | 0.81 | 0.61-1.06 |  |  | 1.12 | 0.91-1.37 |  |
| 20-29 | 1.00 | 0.76-1.31 |  |  | 1.18 | 0.97-1.45 |  |  | 1.00 | 0.77-1.30 |  |  | 1.14 | 0.93-1.40 |  |
| 30-44 | 1.17 | 0.87-1.58 |  |  | 1.05 | 0.84-1.32 |  |  | 1.11 | 0.83-1.47 |  |  | 0.98 | 0.78-1.23 |  |
| ≥ 45 | 1.29 | 0.96-1.73 |  |  | 1.11 | 0.88-1.41 |  |  | 1.19 | 0.89-1.57 |  |  | 1.04 | 0.82-1.32 |  |
| **Treatment: Allergy/Asthma/**  **Lung disease** |  |  | 0.03 |  |  |  | < 0.01 |  |  |  | 0.12 |  |  |  | < 0.01 |
| No | Ref |  |  |  | Ref |  |  |  | Ref |  |  |  | Ref |  |  |
| Yes | 1.25 | 1.02-1.54 |  |  | 1.36 | 1.16-1.59 |  |  | 1.17 | 0.96-1.43 |  |  | 1.29 | 1.10-1.52 |  |
| **Treatment: Immunodeficiency/**  **Transplantation** |  |  | 0.76 |  |  |  | 0.68 |  |  |  | 0.78 |  |  |  | 0.59 |
| No | Ref |  |  |  | Ref |  |  |  | Ref |  |  |  | Ref |  |  |
| Yes | 1.12 | 0.54-2.34 |  |  | 0.89 | 0.51-1.55 |  |  | 1.10 | 0.55-2.21 |  |  | 0.86 | 0.49-1.50 |  |
